# Epidemiology and Hospital Course of Patients With Acute Salpingitis and Coincident HIV

**DOI:** 10.1155/S1064744993000213

**Published:** 1993

**Authors:** Iris Ayala-Rodriguez, Joseph Apuzzio

**Affiliations:** 1 Department of Obstetrics and Gynecology New Jersey Medical School 185 South Orange Avenue Newark NJ 07103-2714 USA

## Abstract

*Objective:* To compare the epidemiology and hospital course of patients with acute salpingitis with and without coincident human immunodeficiency virus (HIV) seropositivity.

*Methods:* Patients admitted to the UMDNJ-University Hospital in Newark, New Jersey from January 1, 1991, to December 31, 1991, with acute salpingitis were studied.

*Results:* Eight percent of all hospitalized patients with acute salpingitis were HIV-positive. The mean age of the HIV-negative group was 25.4 compared with 29.6 years in the HIV-positive group. Gonorrhea and chlamydia were present in 49% and 22%, respectively, in HIV-negatives and in 40% and 20% of HIV-positives. Two of 5 (40%) HIV-positive patients had tuboovarian abscesses compared with 12 of 59 (20%) HIV-negative patients. Three of 5 (60%) HIV-positive patients had admission WBC counts fewer than 10,000/mm^3^ compared to 6 of 59 (12%) of HIV-negatives (*P* = 0.024). The hospital stay was 5.4 days for HIV-positives and 5.8 days for HIV-negatives.

*Conclusions:* Eight percent of hospitalized patients with acute salpingitis were HIV-seropositive. *Neisseria gonorrhoeae* and chlamydia were commonly found organisms in both groups. The initial WBC count was lower for HIV-positive patients. The hospital course of both groups was similar.


Infectious Diseases in Obstetrics and Gynecology 1:91–93 (1993)
© 1993 Wiley-Liss, Inc.

### Epidemiology and Hospital Course of Patients With Acute Salpingitis and Coincident HIV

Iris Ayala-Rodriguez and Joseph Apuzzio
*Department of Obstetrics and Gynecology, New Jersey Medical School, Newark, NJ*

ABSTRACT

***Objective:*** To compare the epidemiology and hospital course of patients with acute salpingitis with and without coincident human immunodeficiency virus (HIV) seropositivity.

***Methods:*** Patients admitted to the UMDNJ-University Hospital in Newark, New Jersey from January 1, 1991, to December 31, 1991, with acute salpingitis were studied.

***Results:*** Eight percent of all hospitalized patients with acute salpingitis were HIV-positive. The mean age of the HIV-negative group was 25.4 compared with 29.6 years in the HIV-positive group. Gonorrhea and chlamydia were present in 49% and 22%, respectively, in HIV-negatives and in 40% and 20% of HIV-positives. Two of 5 (40%) HIV-positive patients had tuboovarian abscesses compared with 12 of 59 (20%) HIV-negative patients. Three of 5 (60%) HIV-positive patients had admission WBC counts fewer than 10,000/mm$^3$ compared to 6 of 59 (12%) of HIV-negatives ($P$ = 0.024). The hospital stay was 5.4 days for HIV-positives and 5.8 days for HIV-negatives.

***Conclusions:*** Eight percent of hospitalized patients with acute salpingitis were HIV-seropositive. *Neisseria gonorrhoeae* and chlamydia were commonly found organisms in both groups. The initial WBC count was lower for HIV-positive patients. The hospital course of both groups was similar.
© 1993 Wiley-Liss, Inc.

KEY WORDS
HIV seropositivity, sexually transmitted diseases

Acute salpingitis is a major cause of both acute and chronic morbidity among young women with the potential for long-term reproductive consequences. Women at risk of acquiring pelvic inflammatory disease (PID) tend to be young women of low socioeconomic background with multiple sexual partners. Human immunodeficiency virus (HIV) infectivity appears to be increasing among similar groups. Sexual risk behaviors are associated with the acquisition of both PID and HIV. Coincident infection with HIV may alter the clinical course of acute salpingitis. The objectives of the study were to determine the seroprevalence and epidemiology of HIV in hospitalized women with acute salpingitis and to compare the hospital course of patients who have acute salpingitis with and without coincident HIV.

### MATERIALS AND METHODS

Charts were reviewed of all women admitted to UMDNJ-University Hospital in Newark, New Jersey, between January 1, 1991, and December 31, 1991, who fulfilled the clinical criteria[1] for the diagnosis of PID. Only those patients with known HIV status were entered in the study.

On the patient's admission, cervical cultures were obtained for *Neisseria gonorrhoeae* and a Chlamydiazyme® (Abbott Laboratories, North Chicago, IL) was performed for chlamydia. Blood was drawn for CBC, SMA18, and venereal disease research labo-

Address correspondence/reprint requests to Dr. Joseph Apuzzio, Department of Obstetrics and Gynecology, New Jersey Medical School, 185 South Orange Avenue, Newark, NJ 07103-2714.

Received April 6, 1993
Accepted June 2, 1993

ratory (VDRL). Fluorescent treponemal antibody test (FTA) was performed if the VDRL was positive. HIV testing was offered after appropriate counseling was given and consent obtained. HIV serologic testing was performed at the University Hospital serology laboratory. Samples positive by enzyme-linked immunosorbent assay (ELISA) were confirmed by Western blot. All patients had pelvic sonograms to detect tuboovarian disease. Antibiotics were prescribed by protocol: Either the combination of cefoxitin, 2 g IV q 6 h, and doxycycline, 100 mg IV q 12 h, or clindamycin, 900 mg IV q 8 h, gentamicin 1.5 mg/kg IV q 8 h, and ampicillin, 2 g IV q 6 h, at the discretion of the admitting physician. Peak and trough serum levels of gentamicin were obtained to adjust the dose of gentamicin. Antibiotics were continued for at least 4 days and at least 48 hours after the patient's defervescence. Upon discharge from the hospital, patients were prescribed oral doxycycline, 100 mg twice a day, for 7 days.

Initial antibiotic therapy was considered a failure if, after 48–72 hours of treatment, the WBC count increased and clinical symptomatology worsened from the initial examination.

Statistical analysis was performed by computer using the Fisher's exact test.

### RESULTS

During the 12 month period from January 1, 1991, to December 30, 1991, 77 patients with acute salpingitis were admitted to University Hospital; 74 charts were available for review. Ten patients were excluded because of unknown HIV status. Of the 64 patients with known HIV status, 5 (8%) were found to be seropositive; no patient had AIDS. The average age of patients who were HIV-negative was 25.4 ± 5.7 years, while those patients who were HIV-positive were all multiparous and older, with a mean age of 29.6 ± 6.3 years. The youngest patient was 18 years old. The demographics are listed in Table 1.

The incidence of positive cervical cultures for gonorrhea and the reactive Chlamydiazyme® as well as VDRL results are listed in Table 2.

All patients with acute salpingitis had ultrasound examinations to document tuboovarian abscesses (TOA). TOA was noted in 2 of 5 (40%) HIV-seropositive patients and in 12 of 59 (20%) HIV-negative patients.

**TABLE 1. Demographics of patients with acute salpingitis**

| Age range (yrs) | HIV (−) (n = 59) (mean age, 25.4−5.7 yrs) [no. (%)] | HIV (+) (n = 5) (8%) (mean age, 29.6 ± 6.3 yrs) |
|---|---|---|
| 15–20 | 13 (22.0) | 1 |
| 21–25 | 18 (30.5) | 0 |
| 26–30 | 18 (20.3) | 1 |
| 31–35 | 7 (11.8) | 2 |
| 36–40 | 2 (3.3) | 1 |
| 41–45 | 1 (1.6) | 0 |

**TABLE 2. STDs among patients with acute salpingitis**

| | HIV status | |
|---|---|---|
| | Positive (n = 5) | Negative (n = 59) |
| *N. gonorrhoeae* | 2 (40%) | 29 (49.1%) |
| Reactive Chlamydiazyme | 1 | 13 (22.0%) |
| Positive VDRL and FTA | 0 | 5 (8.5%) |

Seven patients with TOA initially received cefoxitin and doxycycline. Two of these patients failed initial antibiotic therapy but responded to a change in antibiotics to clindamycin, gentamicin, and ampicillin. One of these patients was HIV-positive. Seven other patients with TOA initially received clindamycin, gentamicin, and ampicillin. Two of these failed initial therapy. One responded when imipenem-cilastatin was prescribed. The other patient required surgical intervention for TOA and experienced a refractory clinical course. She was HIV-negative.

### DISCUSSION

Because of its increasing incidence among women in inner cities, HIV will become more prevalent in the gynecologic setting. Several studies on coincident infection of HIV in patients with acute salpingitis have been published, revealing a 6–17% incidence of HIV positivity among women treated for PID. Hoegsberg et al.[2] in New York found that 14% of inner-city women hospitalized with PID were also HIV-positive. Safrin and co-workers[3] in San Francisco found that 6.7% of women with PID were HIV-positive, and Sperling et al.[4] found 5 of 30 (16.7%) in their population to be HIV-positive.

**TABLE 3. Hospital course of patients with acute salpingitis**

| | HIV status | |
|---|---|---|
| | Positive (n = 5) [no. (%)] | Negative (n = 59) [no. (%)] |
| Admission temp. > 38°C (100.4°F) | 5 (100) | 46 (78) |
| Admission WBC > 10,000/mm$^3$ | 2 (40) | 52 (88.1)* |
| Duration of hospital stay (days) | 5.4 ± 1.02 | 5.8 ± 2.48 |
| Tuboovarian abscess | 2 (40) | 12 (20.3) |
| Surgery | 0 (0) | 1 (1.6) |
| Failed initial antibiotic therapy | 1 (20) | 3 (5.1) |

*$P$ = 0.024

Among our study population of 64 patients admitted with acute salpingitis and known HIV status, 5 of 64 (8%) tested positive for HIV. Perhaps attributable to the small study group, HIV seropositivity did not appear to be associated with a higher frequency of STDs, longer hospital stay, a more refractory clinical course, or an increased rate of surgical intervention (Table 3). The only statistically significant finding was that 3 of 5 (60%) HIV-positive patients had initial WBC counts fewer than 10,000/mm,$^3$ compared to 7 of 59 (12%) HIV-negatives ($P$ = 0.024). The lower initial WBC may be related to a mild immunosuppression of HIV-positive patients. A larger study group is needed to verify this trend in morbidity and the clinical course in HIV-infected women with acute salpingitis compared with women with acute salpingitis who are HIV-negative.

The majority of cases with TOA (13 of 14) responded to IV antibiotics. Seven of the patients with TOA received cefoxitin and doxycycline, with two patients requiring a change of antibiotics after 48 hours of therapy. Seven other patients received triple antibiotics of clindamycin, gentamicin, ampicillin, with two failing initial therapy. One of these patients required surgical intervention for cure.

Because the rate of heterosexual transmission and HIV infectivity among women is increasing, HIV testing should be offered to all patients treated or suspected of having any STD. More importantly, counseling and patient education about STDs and sexual behaviors should assume a greater role in health care practice.

### REFERENCES

1. Hager WD, Eschenbach DA, Spence MR, Sweet RL: Criteria for diagnosis and grading of salpingitis. Obstet Gynecol 61:113–114, 1983.
2. Hoegsberg B, Abulafia O, Sedlis A, Feldman J, DesJalais D, Landesman S, Minkoff H: Sexually transmitted diseases and human immunodeficiency virus infection among women with pelvic inflammatory disease. Am J Obstet Gynecol 163:1135–1139, 1990.
3. Safrin S, Dattel BJ, Hauer L, Sweet RL: Seroprevalence and epidemiologic correlates of human immunodeficiency virus infection in women with acute pelvic inflammatory disease. Obstet Gynecol 75:666–670, 1990.
4. Sperling R, Friedman F Jr, Joyner M, Brodman M, Dottino P: Seroprevalence of human immunodeficiency virus in women admitted to the hospital with pelvic inflammatory disease. J Reprod Med 36:122–124, 1991.
